# Cerium-doped UiO-66-supported Pd catalysts: activity enhancement and deactivation pathways in the carbonylation of methyl nitrite to DMC

**DOI:** 10.1039/d5ra07187a

**Published:** 2026-01-02

**Authors:** Qiuyun Huang, Shiyu Liu, Weihua Shen, Yunjin Fang

**Affiliations:** a State Key Laboratory of Chemical Engineering and Low-Carbon Technology, School of Chemical Engineering, East China University of Science and Technology Shanghai 200237 China whshen@ecust.edu.cn yjfang@ecust.edu.cn +86-21-64252076 +86-21-64252829

## Abstract

For the carbonylation of methyl nitrite (MN) to dimethyl carbonate (DMC), a series of Ce_(*x*)_-UiO-66 were synthesized and utilized as supports for Pd/Ce_(*x*)_-UiO-66 catalysts. The characteristics of the carriers were investigated using XRD, SEM, TG, NH_3_-TPD and BET analyses. The results showed that doping cerium in the UiO-66 significantly enhanced the catalytic activity by improving the surface area, acidity and CO adsorption of the catalysts. Ce_(0.1)_-UiO-66, with the highest thermal stability (547.26 °C), presented the highest catalytic stability in DMC synthesis. It was proven that the damage of C=O and the migration of Pd atoms occurred due to the replacement of ligands and metal clusters, which led to catalytic deactivation.

## Abstract 2

Simultaneously enhancing catalytic performance and investigating the deactivation pathways in DMC synthesis.

## Graphical abstract

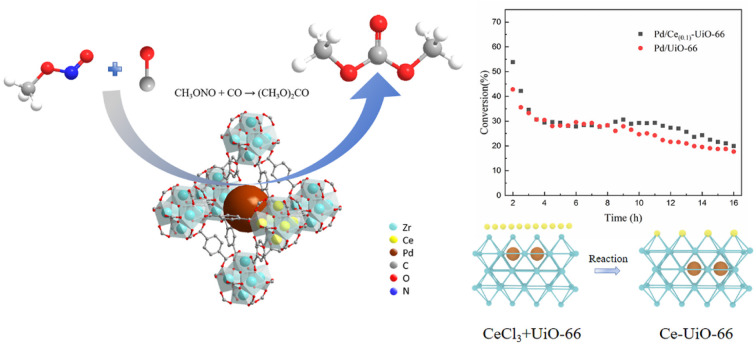

## 1 Introduction

Metal–organic frameworks (MOFs), as novel crystalline porous materials constructed with metal clusters and organic linkers, have drawn much attention in recent years due to their highly adjustable surface area, tunable structure, and periodic porosity.^1–4^ These properties make MOFs promising candidates for diverse applications including gas separation and sorption,^5^ decontamination,^6^ energy storage,^7^ and catalysis.^8,9^ Compared with other MOFs, UiO-66 has outstanding thermal and chemical stability, a large surface area and simple synthesis.^10,11^ Cerium exhibits low toxicity, has a synergistic effect with other active metals, and can easily switch between Ce^3+^ and Ce^4+^.^12^ Ce-UiO-66, which integrates the benefits of Ce and the identical frameworks of UiO-66, has attracted considerable attention in many applications. For example, a Ce-UiO-66-BDC/2,2,6,6-tetramethylpiperidin-1-yl (TEMPO) system was successfully employed for the aerobic oxidation of benzyl alcohol.^13^ Bimetallic Ce–Zr/UiO-66 catalysts were synthesized to degrade chlorinated aromatic hydrocarbons (CAHs) with high catalytic efficiency and less formation of dioxins at a low temperature (100–150 °C).^14^ Ce-UiO-66, with additional active sites and easy regeneration, has also been used for water remediation by efficiently removing contaminants from aqueous media.^15^ Because of its significant catalytic performance in redox reactions and excellent multipollutant adsorption abilities, Ce-UiO-66 has potential in the carbonylation reaction.

Owing to its unique molecular structure, high ionic conductivity and biodegradability, dimethyl carbonate (DMC) has been widely used in the industrial field, including in polycarbonate production,^16^ as a methylation reagent,^17^ as a fuel additive,^18^ as an electrolyte solvent,^19^ and as a pharmaceutical intermediate.^20^ Transesterification,^21^ urea alcoholysis,^22^ methanol oxidative carbonylation^23,24^ and carbon dioxide (CO_2_) direct conversion^25^ have been developed for the industrial synthesis of DMC in the past few years. Among these routes, the indirect oxidation carbonylation of methanol and carbon monoxide (CO) exhibits lower raw-material prices and requires mild reaction conditions, and hence, it is regarded as the most economical and environmentally friendly route. Active carbon,^26^ metal oxides,^27^ zeolites,^28^ and other composite supports^29,30^ have been investigated as supports for Pd-based catalysts for the indirect oxidation carbonylation to DMC. Generally, catalysts can be divided into chlorine-containing and chlorine-free catalysts.^31^ The chlorine in chlorine-containing catalysts can cause damage to the equipment, and these catalysts are easily deactivated due to the loss of chlorine.^32,33^ Meanwhile, chlorine-free catalysts own good catalytic activity and selectivity, with little dimethyl oxalate (DMO) byproduct produced, but the aggregation of Pd clusters also leads to the deactivation of these catalysts.^23^ UiO-66, exhibiting tunable acid sites^34,35^ and a functionalized framework,^36^ is known as an ideal catalyst support for DMC synthesis.

UiO-66 has been studied in the carbonylation of MN to DMC, and previous investigations have focused on modifying UiO-66. For instance, UiO-66 modified with functional groups (X-BDC, X = –NO_2_, –NH_2_, and –CH_3_) has been reported.^34^ Among them, the Pd–NO_2_-UiO-66 exhibited the best catalytic performance, which is attributed to the –NO_2_ group enhancing both the interaction between active species and the support and the adsorption of CO. In another approach, the introduction of TFA as a modulator incorporated Lewis acid sites into UiO-66, and Pd-UiO-66_TFA-0.25_-290 was synthesized.^37^ This catalyst achieved a CO conversion of 68.4%, with a near 100% DMC selectivity based on CO. Moreover, the relationship between the location of active species (Pd NPs) and product selectivity has been demonstrated.^36^ However, cerium-doped UiO-66 has not yet been explored in the carbonylation of MN to DMC, despite its potential structural defects and enhanced adsorption capabilities. Furthermore, the stability of UiO-66 catalysts in continuous heterogeneous reactions and their deactivation processes have rarely been reported.

In this work, Pd/Ce_(*x*)_-UiO-66 (*x* = 0.1, 0.2, 0.5) were synthesized by loading Pd on Ce_(*x*)_-UiO-66 supports, in which cerium was adopted into the UiO-66 frameworks in different contents. The characteristics of the carriers and the catalytic performance of Pd/Ce_(*x*)_-UiO-66 were investigated. The results showed that the incorporation of cerium influenced the crystal size distribution and CO adsorption. With increasing cerium doping content, the thermal stability of Ce_(*x*)_-UiO-66 decreased. The Pd/Ce_(*x*)_-UiO-66 deactivation processes were analyzed. It was proven that the deactivation process occurred in two stages because of the rapid damage of the carboxyl group of the BDC ligands on the surface of the frameworks and the replacement of the metal clusters and organic ligands in the frameworks.

## 2 Experimental

### 2.1 Materials

Zirconium chloride (ZrCl_4_, A.R.), cerium chloride heptahydrate (CeCl_3_·7H_2_O, A.R.), terephthalic acid (H_2_BDC, A.R.), palladium nitrate (Pd(NO_3_)_2_, 39.0 wt% Pd, A.R.), *N*,*N*-dimethylformamide (DMF, A.R.) and glacial acetic acid (CH_3_COOH, A.R.) were purchased from Aladdin Chemical Reagent Co., Ltd. *N*-Hexane (A.R.), acetone (A.R.), and quartz sand (SiO_2_, 50–60 mesh, A.R.) were supplied by Sinopharm Chemical Reagent Co., Ltd. Deionized water (18 MΩ cm) was used throughout the experiments. All chemicals used in this work were purchased from commercial sources and used as received without further purification unless otherwise specified.

### 2.2 Catalyst preparation

#### 2.2.1 Synthesis of UiO-66 and Ce_(*x*)_-UiO-66 carriers

The synthesis of UiO-66 followed the method reported in the literature with some modifications.^5^ ZrCl_4_ (1.50 g, 6.4 mmol) was dissolved in DMF (100 mL, 1.3 mol) at room temperature, followed by 15 minutes of ultrasonic treatment and 3 h of stirring. H_2_BDC (1.07 g, 6.4 mmol) and CH_3_COOH (44 mL, 0.77 mol) were added to the mixture and stirred for 2 h at 70 °C. Deionized water (7.5 mL, 0.42 mol) was then added to the mixture, and it was further stirred for 1 h. The substrate mixture was subsequently placed in a Teflon-lined autoclave, heated for 1 h at 120 °C, and cooled to room temperature. The UiO-66 sample was isolated by centrifugation and washed with DMF and acetone (3 times) before drying under vacuum at 120 °C for 24 h.

The synthesis method of Ce_(*x*)_-UiO-66 (*x* = 0.1, 0.2, 0.5) was similar to that of UiO-66, except that CeCl_3_·7H_2_O was dissolved in deionized water. The combined quantity of ZrCl_4_ and CeCl_3_·7H_2_O was 6.4 mmol, with the molar Ce : Zr ratios of 1 : 10, 1 : 5, and 1 : 1, and the powders were named Ce_(0.1)_-UiO-66, Ce_(0.2)_-UiO-66, and Ce_(0.5)_-UiO-66, respectively.

#### 2.2.2 Synthesis of Pd/UiO-66 and Pd/Ce_(*x*)_-UiO-66 catalysts

The Pd/UiO-66 and Pd/Ce_(*x*)_-UiO-66 catalysts were synthesized using a double-solvent method. Typically, 1.0 g of UiO-66 or Ce_(*x*)_-UiO-66 (*x* = 0.1, 0.2, 0.5) was dispersed in 80 mL of *n*-hexane and was ultrasonically treated for 30 minutes. The suspended mixture was then mixed with 28.7 mg of Pd(NO_3_)_2_, which was previously dissolved in 2 mL of deionized water. After sonicating for 1 h, the precipitates were dried under a vacuum at 80 °C. Finally, the powder was reduced with 10% H_2_/Ar (60 mL min^−1^) at 200 °C for 2 h to obtain the Pd-based catalysts (Pd/UiO-66, Pd/Ce_(0.1)_-UiO-66, Pd/Ce_(0.2)_-UiO-66, and Pd/Ce_(0.5)_-UiO-66).

#### 2.2.3 Synthesis of Pd/UiO-66 + CeCl_3_(*x*) catalysts

The Pd/UiO-66 + CeCl_3_(*x*) catalysts were synthesized by the mechanical mixing of the Pd/UiO-66 with CeCl_3_·7H_2_O. The mass ratios of *m*Pd/UiO-66 to *m*CeCl_3_ were 5 : 1 and 10 : 1, and the samples were named Pd/UiO-66 + CeCl_3_(0.2) and Pd/UiO-66 + CeCl_3_(0.1), respectively.

#### 2.2.4 Synthesis of Pd/UiO-66(2)

The Pd/UiO-66(2) was synthesized by recycling the Pd/UiO-66 after the 10 h reaction using 10% H_2_/Ar (60 mL min^−1^) at 200 °C for 2 h of reduction.

### 2.3 Catalyst characterization

X-ray powder diffraction (XRD) patterns were obtained using a D8 Advance X-ray polycrystalline diffractometer (Bruker, Germany) with a Cu-Kα1 X-ray source.

The Brunauer–Emmett–Teller (BET) surface areas were determined by the N_2_ adsorption–desorption method, which was performed on a Micromeritics-ASAP 2460 instrument (Micromeritics, America).

NH_3_-TPD was performed using a VDSorb-91i programmed temperature chemisorption instrument (Quzhou VODO Instrument Co. Ltd, China). The signal was detected by a thermal conductivity detector. Typically, 0.050 g of the sample was pretreated under a He flow at 120 °C for 2 h to eliminate water and other impurities. The sample was subjected to a flow of 0.5% NH_3_/Ar for 2 h after cooling to 30 °C and held under a He flow for 5 min. The sample was then purged with He until the baseline of the signal became flat. The temperature was raised to 450 °C at a rate of 5 °C min^−1^, and the signal was collected once every second during that period.

CO temperature-programmed desorption (CO-TPD) was conducted on the same test platform as NH_3_-TPD. Typically, 0.050 g of the sample was processed under a He flow for 2 h at 120 °C to get rid of impurities. After cooling to 30 °C, it was placed under a He flow for 5 min. A 10% H_2_/Ar gas mixture was then introduced into the reactor at 200 °C for 2 h. The sample was treated with a flow of 5% CO/Ar for 2 h after cooling to 30 °C and held under He for 5 min. The sample was then purged with He until the baseline of the signal became flat. At a heating rate of 5 °C min^−1^, the CO-TPD profiles were obtained in the 30–450 °C range.

Transmission electron microscopy (TEM) and energy-dispersive spectroscopy (EDS) observations of the catalysts were carried out on an AFM-Raman-SEM instrument.

Scanning electron microscopy (SEM) images of the samples were collected using a Nova Nano SEM 450 instrument (FEI Instrument Co., America).

Thermogravimetric analysis (TGA) data were obtained using a TGA 8000 instrument (PerkinElmer, America).

Inductively coupled plasma optical emission spectrometry (ICP-OES) was performed using a 5800 ICP-OES instrument (Agilent, US).

Fourier transform infrared (FTIR) spectroscopy was carried out using a Nicolet 6700 instrument (Thermo Electron, America).

X-ray photoelectron spectroscopy (XPS) of the catalysts was performed using an Escalab 250Xi spectrometer (Thermo Fisher, America) equipped with an X-ray source of Al-Kα (1486.7 eV), and the binding energy was referenced to the C 1s peak at 284.8 eV.

### 2.4 Catalyst evaluation

In this work, MN was prepared *in situ*. Eqn (1) illustrates the MN synthesis reaction. The compound was produced by the reaction of oxygen (from air) with NO and methanol in a bubble column. Through condensation in the two coolers set at room temperature and −3 °C, water and methanol in the outlet gas were eliminated, respectively. The experiment was performed on a fixed-bed stainless-steel tube reactor connected to an online gas chromatograph (GC 2060, Ruimin, China), which included a thermal conductivity detector and a flame ionization detector. The reactions of MN carbonylation to the DMC product and DMO byproduct are shown in eqn (2) and (3), respectively. The reaction conditions were 120 °C and 0.1 MPa, and the gas mixture of CO/MN/Ar/N_2_/NO (5/13.5/10/60/3) was fed into the reactor loaded with 0.5 g of the catalyst mixed with 0.5 g of quartz sand.

The reaction equations included in eqn (1)–(3) are shown below:

1 4NO + 4CH_3_OH + O_2_ = 4CH_3_ONO + 2H_2_O

2 2CH_3_ONO + CO = (CH_3_O)_2_CO + 2NO

3 2CH_3_ONO + 2CO = (COOCH_3_)_2_ + 2NO

The conversion of CO and the selectivity of DMC based on CO were calculated using the following formulae:

4 Conversion of CO (%) = (*n*(CO)_in_ − *n*(CO)_out_)/*n*(CO)_in_ × 100%

5 Selectivity of DMC based on CO (%) = *n*(DMC)/(*n*(DMC) + 2 × *n*(DMO)) × 100%

## 3 Results and discussion

**Fig. 1:**
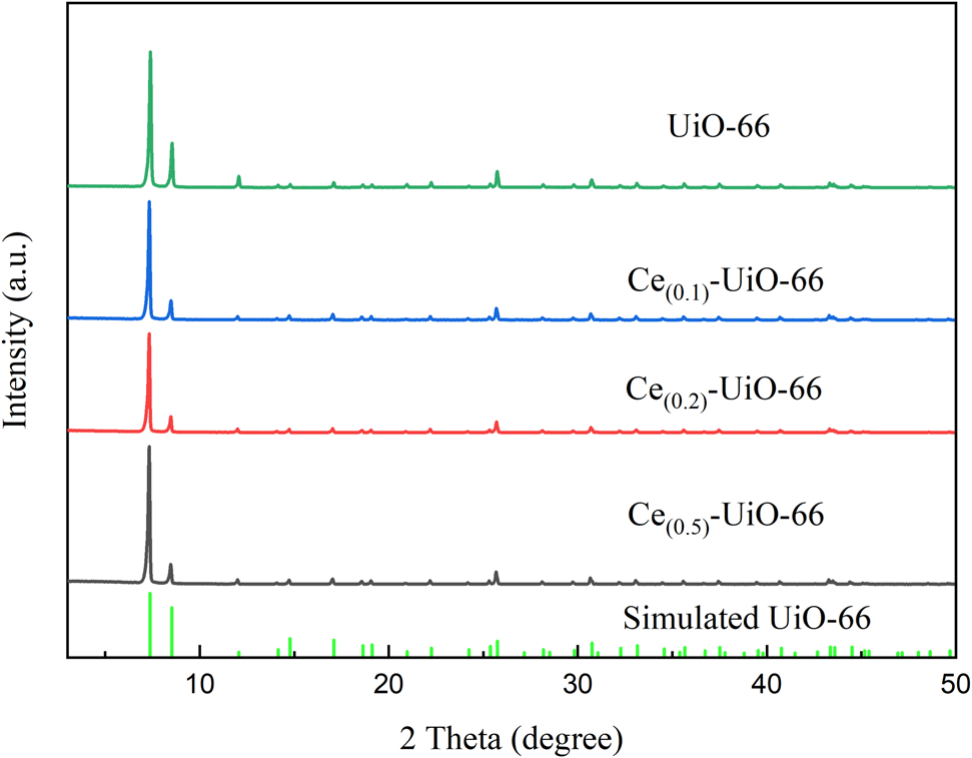
XRD patterns of Ce_(0.1)_-UiO-66, Ce_(0.2)_-UiO-66, Ce_(0.5)_-UiO-66 and UiO-66.

The XRD patterns of the Ce_(*x*)_-UiO-66 and UiO-66 are displayed in Fig. 1. The crystal structures of these samples matched the simulated UiO-66 patterns, indicating that the incorporation of cerium had little impact on the crystallization of the UiO-66 units.

**Fig. 2:**
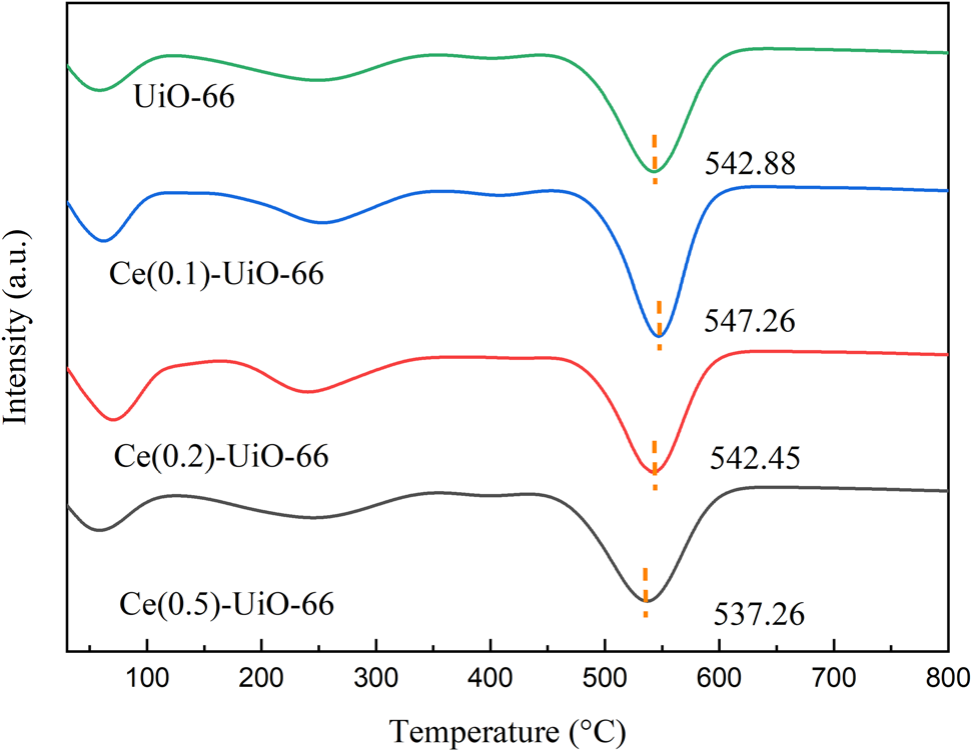
DTG curves of Ce_(0.1)_-UiO-66, Ce_(0.2)_-UiO-66, Ce_(0.5)_-UiO-66 and UiO-66 under a nitrogen flow.

The thermal stability of the Ce_(*x*)_-UiO-66 and UiO-66 samples was investigated by TGA, as shown in Fig. 2. According to Fig. S1, all of the samples had comparable TG curves and retained the majority of their weight below 450 °C. The evaporation of physically adsorbed water was responsible for the weight loss observed between 50 °C and 150 °C, whereas the peak at 250 °C was attributed to the removal of DMF and structurally coordinated water.^38^ The sharp weight loss that occurred after 450 °C represented the collapse of frameworks and the decomposition of the organic ligands.^39^ It was obvious that the Ce_(0.1)_-UiO-66 and Ce_(0.5)_-UiO-66 exhibited the highest and lowest collapse temperatures of 547.26 °C and 537.26 °C, respectively, and with increasing cerium doping content, the thermal stability of the frameworks decreased. In addition, Ce_(0.1)_-UiO-66 had a higher collapse temperature than UiO-66 (542.88 °C). This suggests that cerium had a synergistic effect with zirconium, and doping cerium in the UiO-66 frameworks with zirconium created a new phase, which enhanced the thermal stability of the sample.^5,15^ Moreover, higher contents of cerium hampered the integrity of the frameworks, which led to decreased thermal stability.

**Fig. 3:**
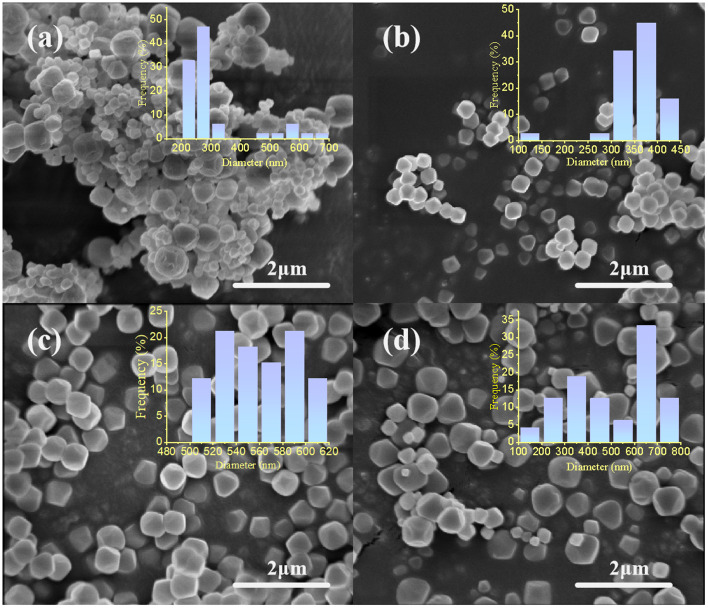
The SEM images of (a) UiO-66, (b) Ce_(0.1)_-UiO-66, (c) Ce_(0.2)_-UiO-66 and (d) Ce_(0.5)_-UiO-66.

The SEM images of Ce_(*x*)_-UiO-66 and UiO-66 are presented in Fig. 3, which assesses the morphological influence of Ce doping on the UiO-66. The distribution of the Ce_(*x*)_-UiO-66 and UiO-66 particles varied with the incorporation amount of Ce. The UiO-66 exhibited a wide particle size distribution, ranging from 200 nm to 700 nm. The majority of the crystal size was centered in the range of 200–300 nm, with only a small percentage of particles having a size close to 575 nm. After doping Ce on the UiO-66, the distribution range of the crystal size was significantly narrowed, with the concentrated size of 300–450 nm for Ce_(0.1)_-UiO-66 and 500–620 nm for Ce_(0.2)_-UiO-66. Besides, despite the increase in the crystal size, the yield of the samples decreased with the increased increasing molar ratio of Ce : Zr. Typically, under the same synthesis conditions and at the same precursor solution volume as those mentioned before, the resulting amount of Ce_(0.5)_-UiO-66 was 0.87 g, while that of UiO-66 was 1.85 g. It can be inferred that the incorporation of Ce inhibited the nucleation process of crystals, thus leading to the increase in the crystal size and the reduction in the yield of the samples.

The BET surface areas of the Ce_(*x*)_-UiO-66 and UiO-66 samples are shown in Table S1. A significant increase in the specific surface area of Ce_(*x*)_-UiO-66 was observed after the doping of cerium in UiO-66. As the molar ratio of Ce : Zr increased from 0 to 1, the surface area of the corresponding sample increased from 505.35 m^2^ g^−1^ to 917.55 m^2^ g^−1^. The dramatic increase in the surface area of Ce_(*x*)_-UiO-66 could be attributed to the missing linker defects generated through Ce doping.^13^

**Fig. 4:**
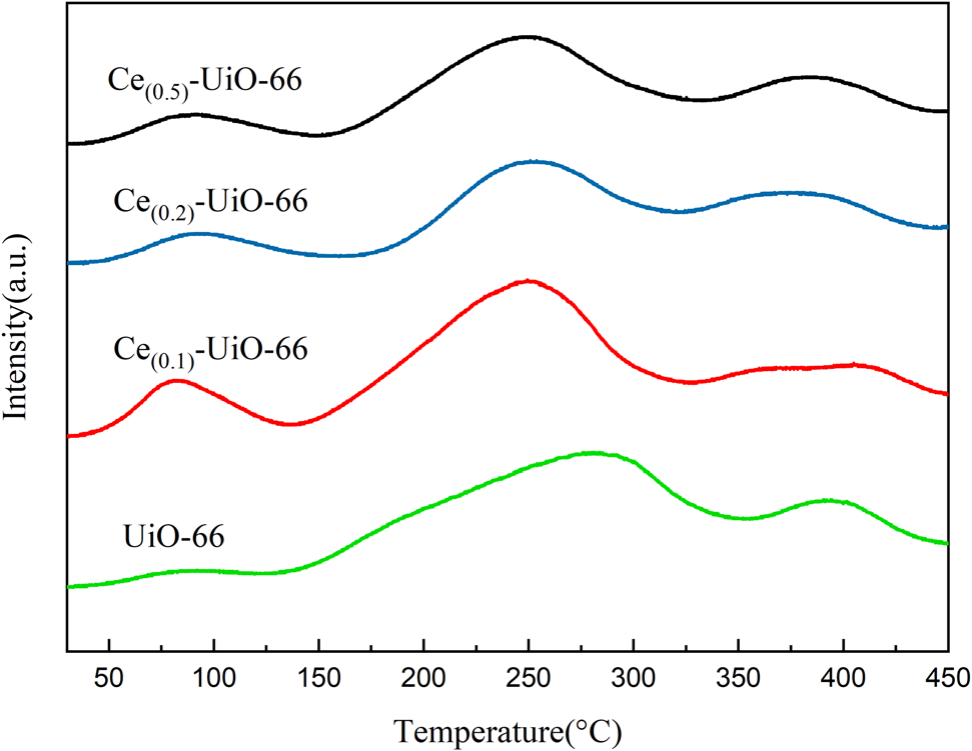
NH_3_-TPD results of Ce_(0.1)_-UiO-66, Ce_(0.2)_-UiO-66, Ce_(0.5)_-UiO-66 and UiO-66.

**Fig. 5:**
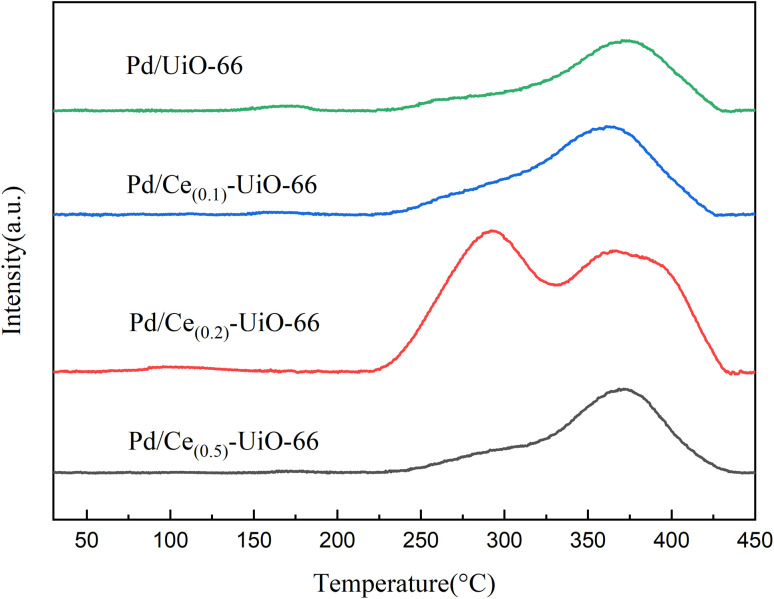
CO-TPD results of Pd/Ce_(0.1)_-UiO-66, Pd/Ce_(0.2)_-UiO-66, Pd/Ce_(0.5)_-UiO-66 and Pd/UiO-66.

NH_3_-TPD and CO-TPD were employed to investigate the acid sites of the carriers and the CO adsorption property of these catalysts, respectively. As shown in Fig. 4, there were three desorption peaks in the range of 30–450 °C for all the samples. The doping of cerium increased the intensity of the first peak between 50 and 130 °C, which represents the weak acid sites. Compared with UiO-66, the second peaks of other samples, which represent the medium acid sites, were shifted to a lower temperature with the doping of cerium, indicating that the acidity of the medium acid sites of Ce_(*x*)_-UiO-66 weakened. In addition, a new peak was observed for Ce_(0.1)_-UiO-66, which could be attributed to the synergistic effect between Ce and Zr that enhanced the acidity of strong acid sites. Fig. 5 shows the CO desorption peaks in the range of 200–450 °C for all the catalysts. The intensity of the desorption peak at about 290 °C was also observed to strengthen for Pd/Ce_(0.1)_-UiO-66 and Pd/Ce_(0.2)_-UiO-66, indicating that cerium doping in the UiO-66 enhanced the interaction between CO and the surface of the catalysts.

**Fig. 6:**
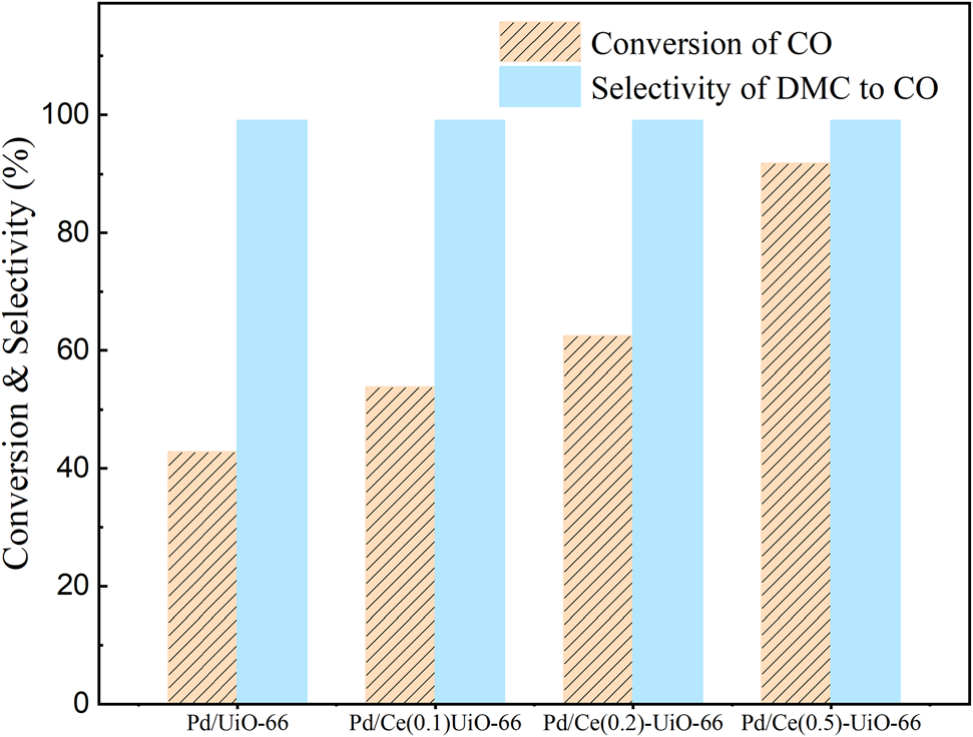
The conversion of CO and the selectivity of DMC to CO in 2 h on Pd/UiO-66, Pd/Ce_(0.1)_-UiO-66, Pd/Ce_(0.2)_-UiO-66 and Pd/Ce_(0.5)_-UiO-66. Reaction conditions: CO/MN/Ar/N_2_/NO = 5/13.5/10/60/3, 120 °C, and 0.1 MPa.

**Fig. 7:**
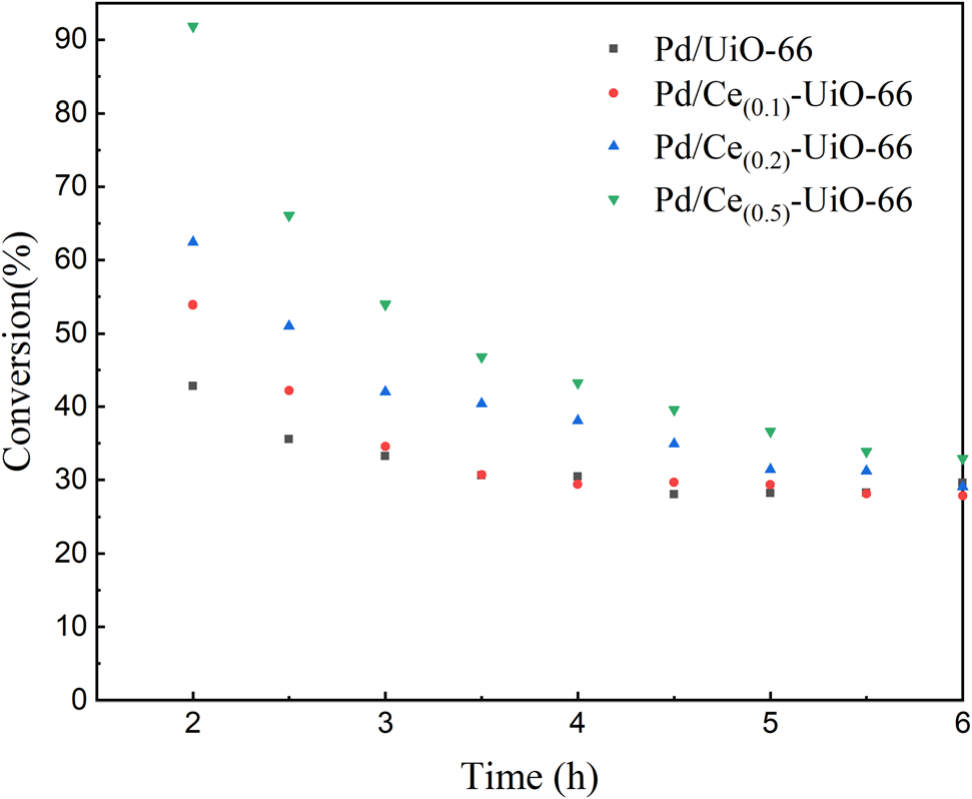
The CO conversion during the 6 h reaction on Pd/UiO-66, Pd/Ce_(0.1)_-UiO-66, Pd/Ce_(0.2)_-UiO-66 and Pd/Ce_(0.5)_-UiO-66. Reaction conditions: CO/MN/Ar/N_2_/NO = 5/13.5/10/60/3, 120 °C, and 0.1 MPa.

To investigate the influence of cerium incorporation on the UiO-66, the catalytic performance of the Pd/UiO-66 and Pd/Ce_(*x*)_-UiO-66 catalysts for MN carbonylation to DMC was explored, and the results are shown in Fig. 6, 7 and S2. It is suggested that cerium had a positive effect on the catalytic activity in the reaction. Among these catalysts, the Pd/Ce_(0.5)_-UiO-66 exhibited the highest conversion of CO, and the conversion decreased from 91.8% (2 h) to 32.9% (6 h). Besides, all the catalysts had a superior DMC selectivity based on CO (>99%), which was due to the high dispersion of Pd, as confirmed by XRD results. The selectivity of DMC based on MN of the catalysts was 83% (Fig. S2), and the byproducts formed were methyl formate, methanol and dimethoxymethane.^40^Fig. 7 shows that the CO conversion of all the catalysts decreased and eventually tended to the same value. Based on preliminary tests indicating the superior stability of Pd/Ce_(0.1)_-UiO-66 among the cerium-doped catalysts, experiments on the Pd/UiO-66 and this catalyst were performed for 16 h in order to further elucidate the impact of cerium doping on the catalyst stability, and the results are shown in Fig. S3. The CO conversion of Pd/UiO-66 decreased at the beginning of the reaction, remained constant after 4 h of reaction, and then continued to decline again after 8 h of the reaction. After doping cerium in the frameworks, the time for which the conversion occurred was prolonged, and the second inactivation of the Pd/Ce_(0.1)_-UiO-66 occurred after 11 h of the reaction. This suggested that doping cerium into the UiO-66 frameworks improved the stability of the catalyst. It was evident that during the reaction, both catalysts exhibited two distinct deactivation curves: an initial rapid CO conversion decline phase, followed by a linear deactivation phase, indicating the presence of two deactivation processes.

A series of analyses was conducted to deeply examine the mechanisms of the deactivation processes. The XRD patterns of the Pd/Ce_(0.1)_-UiO-66 after different reaction periods (Fig. S4) exhibited the same diffraction peaks with little intensity differences, revealing that the supports maintained a good crystalline structure throughout the Pd loading and reaction processes. Moreover, the crystal structures of Pd/Ce_(0.1)_-UiO-66, Pd/Ce_(0.2)_-UiO-66 and Pd/Ce_(0.5)_-UiO-66 remained unchanged, and no Bragg peaks corresponding to PdO (JCPDS no. 41-1107) or Pd metal (JCPDS no. 01-1201) were observed, indicating that the Pd species exhibited a high dispersion on the supports. The BET results of the Pd/Ce_(0.1)_-UiO-66 catalyst after the reaction were similar to those of the fresh Pd/Ce_(0.1)_-UiO-66 catalyst (Table S2), which also suggested that the frameworks of the catalysts maintained their specific surface area, pore size and volume.

**Fig. 8:**
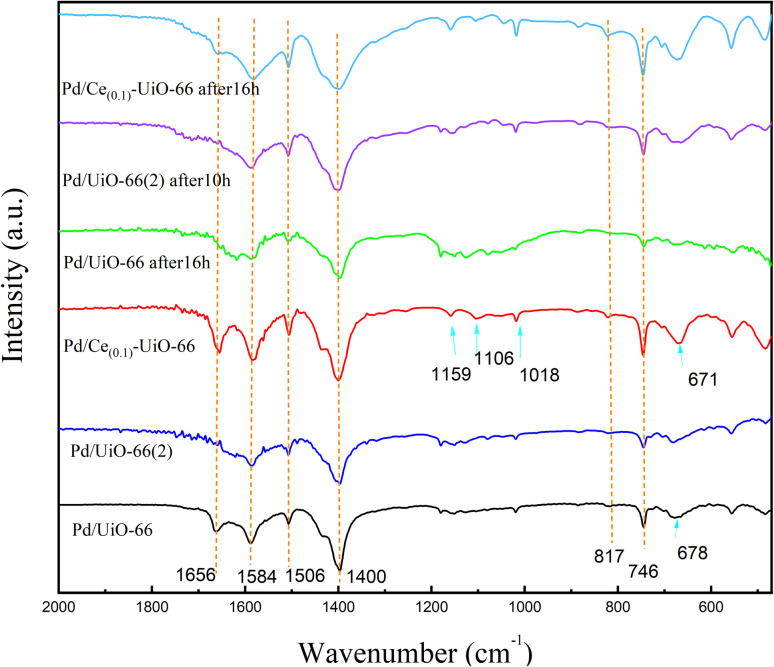
FTIR spectra of the Pd/UiO-66, Pd/UiO-66(2), and Pd/Ce_(0.1)_-UiO-66 catalysts before and after the reaction.

The FTIR spectra of Pd/UiO-66, Pd/UiO-66(2), and Pd/Ce_(0.1)_-UiO-66 catalysts before and after the reaction are shown in Fig. 8. For all the samples, the bands at 1584 cm^−1^ and 1400 cm^−1^ were attributed to the carboxylic acid groups of BDC, arising from the O–C–O anti-symmetric and symmetric stretching vibrations, respectively.^5,41^ And the band at 1506 cm^−1^ was assigned to the C=C vibration of the benzene ring.^14^ The bands at 817 cm^−1^ and 746 cm^−1^ were linked to the C–H vibration of BDC ligands.^38^ These peaks retained their intensity during the reaction, indicating that the corresponding structure remained stable during the reaction. Moreover, the bands at 1159 cm^−1^, 1106 cm^−1^, and 1018 cm^−1^ appearing in Pd/Ce_(0.1)_-UiO-66 were attributed to the C–O stretching vibration, and the band at 678 cm^−1^ in Pd/UiO-66 was linked to the O–H vibration of the BDC ligands, which moved to 671 cm^−1^ in Pd/Ce_(0.1)_-UiO-66. These changes observed for Pd/Ce_(0.1)_-UiO-66 revealed the enhanced stability of the corresponding structure; thus, cerium was successfully doped in UiO-66. Only the band at 1656 cm^−1^ in Pd/UiO-66 and Pd/Ce_(0.1)_-UiO-66 catalysts, which represented the C=O carbonyl stretching vibrations of the BDC ligands,^42^ disappeared in the catalysts after the reaction, suggesting that the structure was damaged.

**Fig. 9:**
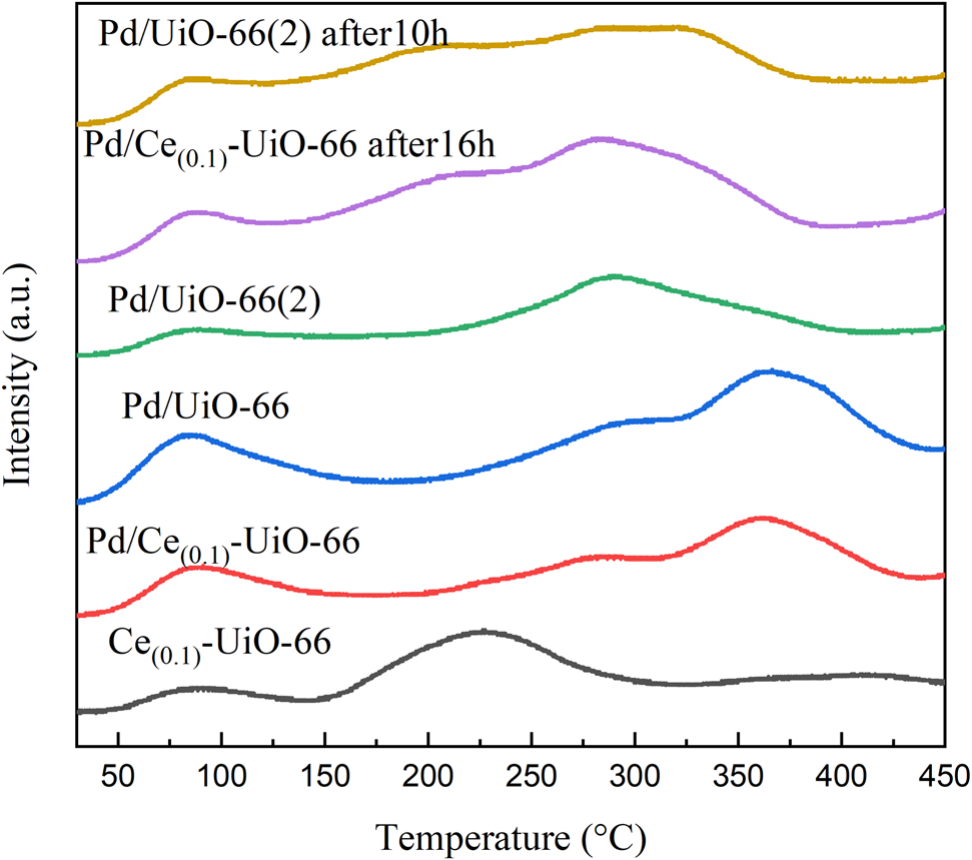
NH_3_-TPD profiles of Pd/UiO-66(2) and Pd/Ce_(0.1)_-UiO-66 catalysts (both fresh and after reaction), Ce_(0.1)_-UiO-66, and Pd/UiO-66.

The NH_3_-TPD results of Pd/UiO-66(2) and Pd/Ce_(0.1)_-UiO-66 catalysts before and after the reaction, Ce_(0.1)_-UiO-66, and Pd/UiO-66 are shown in Fig. 9. Compared with Ce_(0.1)_-UiO-66, the acid strength of the samples changed significantly after the Pd loading process. Two peaks between 200 °C and 425 °C appeared after this process, corresponding to medium acid sites, revealing the increased acidity of the Pd/Ce_(0.1)_-UiO-66. Similar to the carriers (Fig. 4), the second peaks of the Pd/UiO-66, which represented medium acid sites, were moved to a lower temperature in Pd/Ce_(0.1)_-UiO-66, which indicated that the acidity of medium acid sites in Pd/Ce_(0.1)_-UiO-66 also weakened. For Pd/UiO-66(2) and Pd/Ce_(0.1)_-UiO-66 after 10 h and 16 h of the reaction, it was observed that the peaks after the reaction at about 370 °C, which represent the Brønsted acid sides, disappeared,^35^ while the intensity of the peaks between 125 °C and 400 °C increased. Connecting with the FTIR spectra, where the band corresponding to the carbonyl stretching vibrations of the BDC ligands disappeared after the reaction, it can be inferred that strong acid sites were formed by the carboxyl group of BDC ligands and damaged during the 6 h reaction. The strong acid sites of the catalyst could hardly be recovered, as there were no peaks belong to the strong acid sites in the NH_3_-TPD results of Pd/UiO-66(2) and Pd/UiO-66 after 10 h and 16 h of the reaction. These changes might be the reason for the first catalytic deactivation process. The results in Fig. S5 show that the CO conversion on the Pd/UiO-66(2) after 1 h was close to the conversion on Pd/UiO-66 after 10 h, which was consistent with the NH_3_-TPD results. Furthermore, the peaks observed at around 225 °C for UiO-66 appeared again in the samples after the reaction, indicating that the structure that created the acid sites of the carriers was recovered during the reaction. It is proposed that the replacement of the metal clusters and organic ligands in the frameworks might have occurred, which led to the second deactivation process.

From the FTIR and NH_3_-TPD results and two stages of deactivation curves observed, it was concluded that two deactivation processes occurred during the reaction. The hypothesis to explain the two deactivation processes is presented. The high conversion of CO might be caused by the high acidity of the carrier. After the damage of the carboxyl group of the BDC ligands on the surface of the frameworks, the catalyst deactivated rapidly. Besides, the crystallization process was dynamic and reversible, the ligands and metal clusters were replaceable with each other, and this exchange occurred between the ligands and metal clusters on the UiO-66 frameworks during the reaction. The Pd atoms migrated from the surface to the interior of the carrier with the replacement of the ligands and metal clusters in the interlayers of the frameworks, resulting in their more uniform distribution. Due to the migration of Pd particles into the interior of the frameworks, on the one hand, the internal diffusion of reactants into the interior pores of the framework was restricted, which inhibited their reaction with the active center; on the other hand, Pd particles might have moved to inactive sites during the migration process, resulting in the steady deactivation of the catalyst. For the first deactivation curve, both deactivation processes occurred, and the damage of the strong acid sites was the dominant factor. For the second deactivation curve, the carboxyl group of the catalysts was damaged, and the migration of Pd atoms led to catalyst deactivation. In addition, the stability of Pd/Ce_(0.1)_-UiO-66 was further evaluated over 70 h, and the observed deactivation behavior was consistent with the above discussion (see the SI for details). Consistently, the STEM and EDS characterizations of the catalyst before and after the reaction further supported the occurrence of Pd migration and aggregation (Fig. S10–S12).

As cerium can be doped into the frameworks of UiO-66 without damaging the crystalline structure, the hypothesized deactivation mechanism for the second process would be validated, if the doping of cerium into the frameworks occurs during the reaction, when the physical mixing of CeCl_3_ with UiO-66 reacts under the same conditions.

**Scheme 1:**
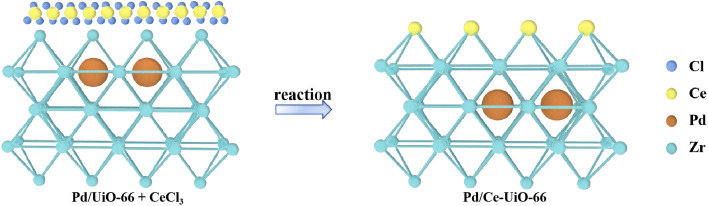
Diagram of the reaction process of the Pd/UiO-66 + CeCl_3_ catalyst.

**Fig. 10:**
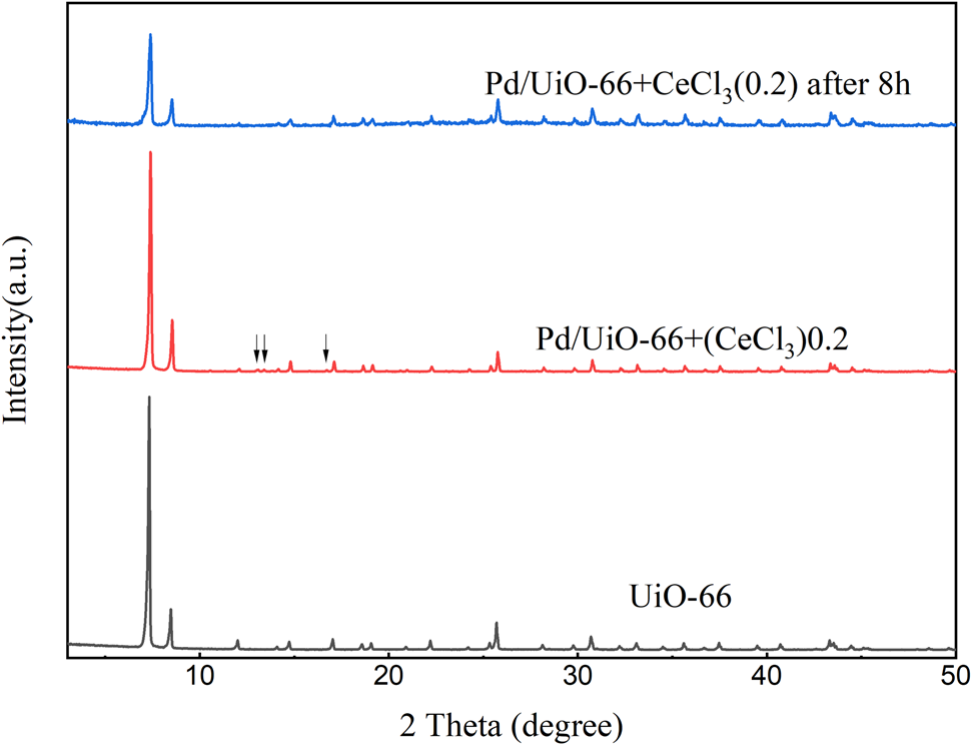
XRD patterns of UiO-66, Pd/UiO-66 + CeCl_3_(0.2) and Pd/UiO-66 + CeCl_3_(0.2) after the 8 h reaction.

To confirm this conjecture, we experimented under the same reaction conditions using the Pd/UiO-66 + CeCl_3_(0.2) catalyst (Scheme 1), in which cerium chloride was mixed with the fresh Pd/UiO-66 catalyst (*m*CeCl_3_ : *m*Pd/UiO-66 = 1 : 5 wt%). The results showed that the Pd/UiO-66 + CeCl_3_(*x*) catalysts, in which CeCl_3_ was mechanically mixed with Pd/UiO-66, exhibited a similar catalytic performance to Pd/Ce_(*x*)_-UiO-66, which increased as the cerium content increased (Fig. S13). The XRD patterns of the fresh catalyst and the catalyst after the 8 h reaction are shown in Fig. 10. Compared with the simulated UiO-66 crystal structure, there were three new peaks in the fresh catalyst that was mixed with cerium, and these peaks disappeared after the 8 h reaction. It is confirmed that the crystallization process with the replacement of the ligands and metal clusters happened, and cerium participated in the formation of the frameworks.

**Fig. 11:**
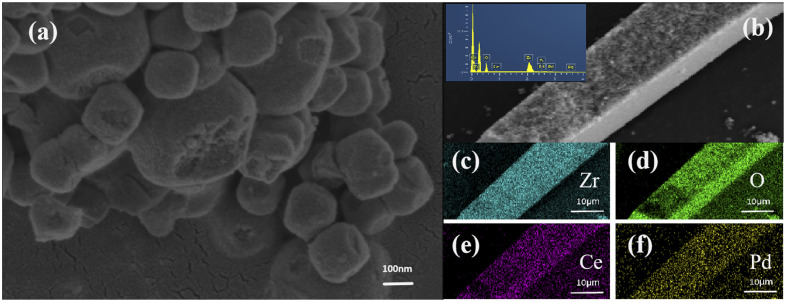
(a and b) STEM images of Pd/UiO-66 + CeCl_3_(0.2) after the 8 h reaction and Pd/UiO-66 + CeCl_3_(0.2) after the 8 h reaction and washing and (c–f) EDS elemental mappings of Zr, O, Ce, and Pd in Pd/UiO-66 + CeCl_3_(0.2) after the 8 h reaction and washing.

Due to the high solubility of CeCl_3_ in ethanol, the CeCl_3_ in the catalyst was removed by washing the sample with ethanol three times, and the sample was dried at 120 °C for 24 h under vacuum. The morphology of the resulting powder and the distribution of different species were characterized by STEM and EDS, respectively. As shown in Fig. 11, numerous smaller particles were observed inside the large crystal particles. The presence of cerium and zirconium with uniform distributions on the surface of the sample was confirmed by the EDS image, which indicated that cerium participated in the formation of the frameworks, thus confirming the hypothesis of the deactivation mechanism of the second phase.

**Fig. 12:**
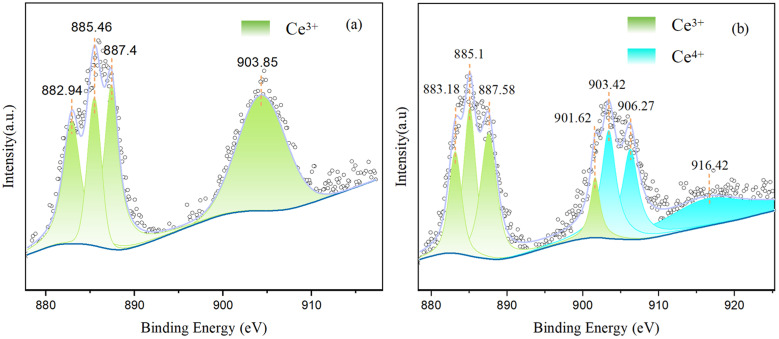
XPS spectra of (a) Pd/UiO-66 + CeCl_3_(0.2) and (b) Pd/UiO-66 + CeCl_3_(0.2) after the 8 h reaction.

The chemical states of the elements before the reaction and the valence states of different chemical species after the reaction for the Pd/UiO-66 + CeCl_3_(0.2) catalyst were determined by XPS. The XPS spectra illustrated the presence of Ce, Zr, O, C, and Pd elements in these samples (Fig. S14). The deconvolution of the high-resolution XPS spectra of Ce 3d is shown in Fig. 12. The four peaks at 882.94 eV, 885.46 eV, 887.4 eV, and 903.85 eV indicated that only Ce^3+^ existed in the sample before the reaction (Fig. 12a). Besides, the peaks at 883.18 eV, 885.1 eV, 887.58 eV, and 901.62 eV corresponded to the Ce^3+^, and the new peaks at 903.42 eV, 906.37 eV, and 916.42 eV were related to Ce^4+^ (Fig. 12b). The new dual-oxidation states of Ce^3+^ and Ce^4+^ in the catalyst after the reaction revealed that during the reaction process, the cerium species were involved in the composition of the frameworks. In addition, with the peaks that were present in the Pd/UiO-66 + CeCl_3_(0.2) catalysts, no characteristic diffraction peaks for Ce 3d were observed in the Ce_(0.2)_-UiO-66 samples, which could be a result of the highly dispersed cerium on the surface of the frameworks. As the CeCl_3_ powder was mechanically mixed with the Pd/UiO-66 power, the cerium species exhibited a higher concentration on the surface of the Pd/UiO-66 + CeCl_3_(0.2) sample, which made them easier to detect by XPS.

## 4 Conclusion

In summary, a series of Ce_(*x*)_-UiO-66 (*x* = 0.1, 0.2, 0.5) was synthesized and used as supports for Pd/Ce_(*x*)_-UiO-66 catalysts for MN carbonylation to DMC. The results showed that cerium was successfully incorporated in the UiO-66 frameworks without destroying the crystallization, it enhanced the surface area, and it influenced the crystal size distribution and thermal stability. The Pd/Ce_(*x*)_-UiO-66 catalysts demonstrated enhanced catalytic performance in the carbonylation of MN to DMC, and the activity improved with the cerium doping content. The catalysts were involved in two stages of deactivation processes, which were due to the damage of the C=O structure in BDC ligands and Pd migration from the surface into the UiO-66 framework interior, which was driven by the replacement of the ligands and metal clusters within the frameworks. This work systematically revealed the influence of cerium doping in UiO-66 frameworks and the deactivation processes of Pd/Ce_(*x*)_-UiO-66 in DMC synthesis in a fixed-bed reactor.

## Conflicts of interest

The authors declare that they have no known competing financial interests or personal relationships that could have appeared to influence the work reported in this paper.

## Supplementary Material

RA-016-D5RA07187A-s001

## Data Availability

The authors confirm that the data supporting the findings of this study are available within the article. Supplementary information (SI) is available. See DOI: https://doi.org/10.1039/d5ra07187a.
