# Supplementary material for: Cerium-doped UiO-66-supported Pd catalysts: activity enhancement and deactivation pathways in the carbonylation of methyl nitrite to DMC
Source: RSC Adv. 2026 Jan 2;16(2):1202–11. doi: 10.1039/d5ra07187a (PMC12758001; doi:10.1039/d5ra07187a)
Supplement: RA-016-D5RA07187A-s001 [file RA-016-D5RA07187A-s001.pdf]

Supplementary Information (SI) for RSC Advances.
This journal is © The Royal Society of Chemistry 2025

Supporting Information

Cerium-Doped UiO-66 Supported Pd Catalysts: Activity Enhancement and Deactivation Pathways in Carbonylation of methyl nitrite to DMC

Qiuyun Huang, Shiyu Liu, Weihua Shen*, Yunjin Fang*

State Key Laboratory of Chemical Engineering, School of Chemical Engineering, East China University of Science and Technology, Shanghai 200237, China

Weihua Shen*: whshen@ecust.edu.cn

Tel: +86-21-64252076

Yunjin Fang*: yjfang@ecust.edu.cn

Tel: +86-21-64252829

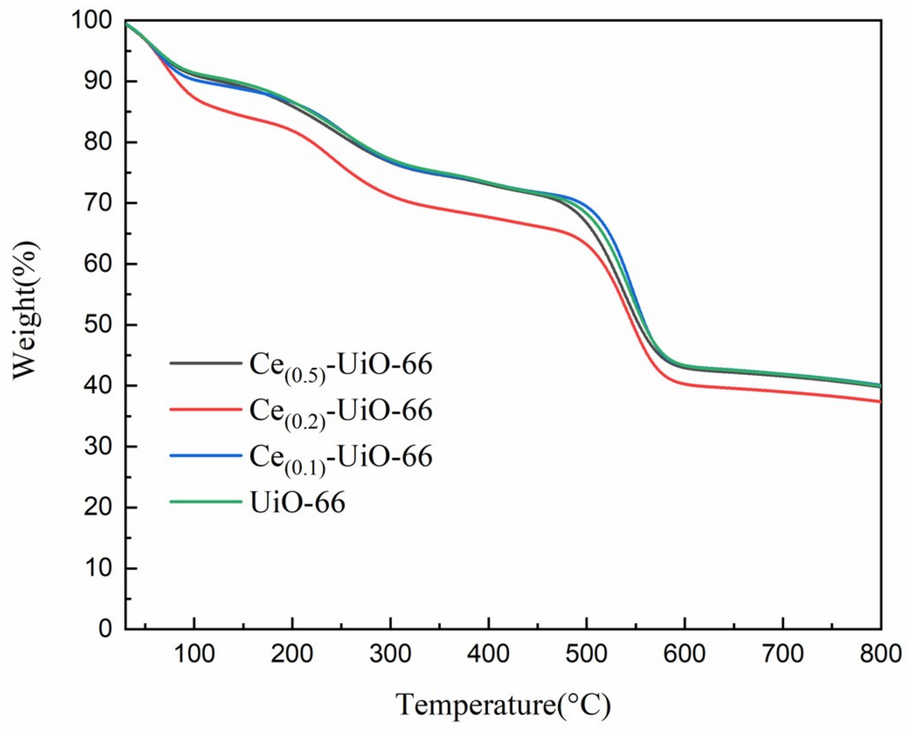

Fig. S1. TG curves under nitrogen flow of $Ce_{(0.1)}$-UiO-66, $Ce_{(0.2)}$-UiO-66, $Ce_{(0.5)}$-UiO-66 and UiO-66.

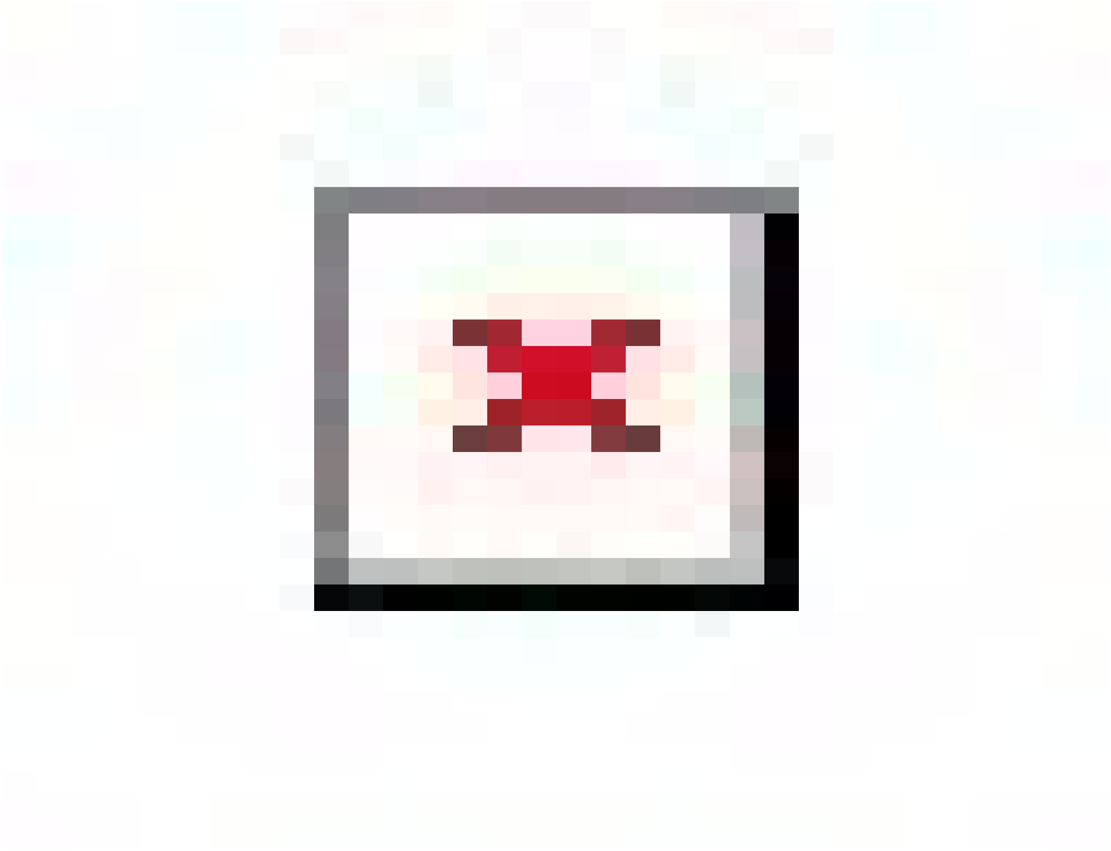

Fig. S2. The conversion of MN and the selectivity of DMC to MN in 2 h of Pd/UiO-66, Pd/$Ce_{(0.1)}$-UiO-66, Pd/$Ce_{(0.2)}$-UiO-66 and Pd/$Ce_{(0.5)}$-UiO-66. Reaction conditions: CO/MN/Ar/N_2/NO =5/13.5/10/60/3, 120˚C, 0.1 MPa.

The equations of the side reactions are shown below:

$$4CH_3ONO = CHOOCH_3 + 2\ CH_3OH + 4\ NO \qquad (S1)$$

$$2\ CH_3ONO + CH_3OH = (CH_3O)_2CH_2 + H_2O + 2\ NO \qquad (S2)$$

The conversion of MN and the selectivity of DMC based on MN were calculated using the following formulae:

$$\text{Conversion of MN (\%)} = (n(MN)_{in} - n(MN)_{out}) / n(MN)_{in} \times 100\% \qquad (S3)$$

$$\text{Selectivity of DMC based on MN (\%)} = 2n(DMC) / (n(MN)_{in} - n(MN)_{out}) \times 100\% \qquad (S4)$$

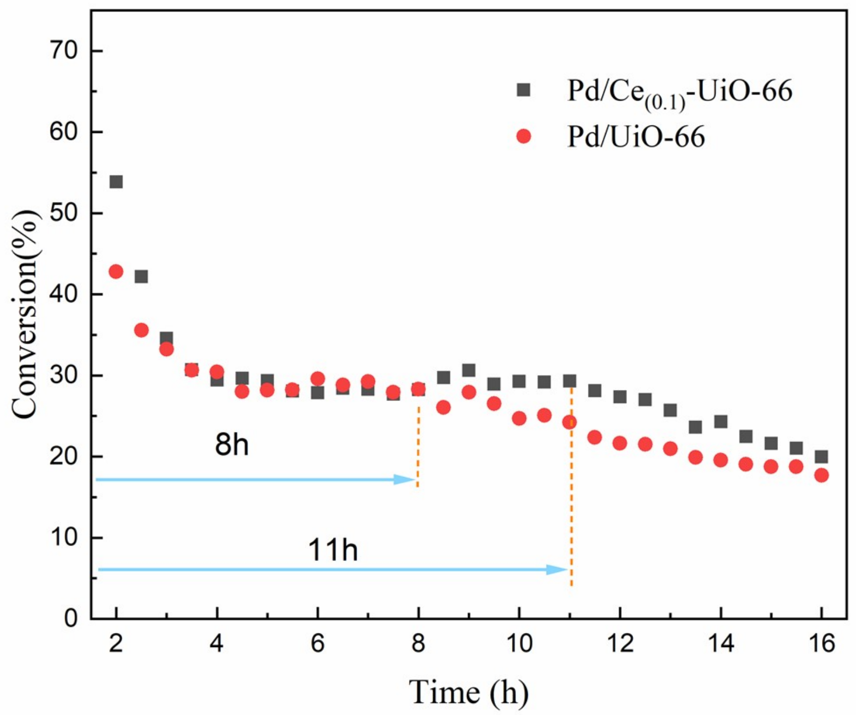

Fig. S3. The CO conversion during the 16 h reaction of Pd/UiO-66 and Pd/$Ce_{(0.1)}$-UiO-66. Reaction conditions: CO/MN/Ar/N_2/NO =5/13.5/10/60/3, 120˚C, 0.1 MPa.

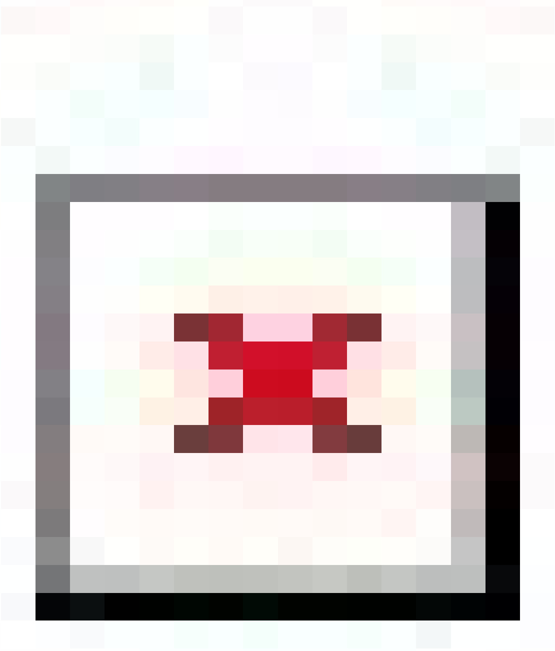

Fig. S4. XRD patterns of $Ce_{(0.1)}$-UiO-66, Pd/$Ce_{(0.1)}$-UiO-66, Pd/$Ce_{(0.1)}$-UiO-66 after 8 h reaction, Pd/$Ce_{(0.2)}$-UiO-66 and Pd/$Ce_{(0.5)}$-UiO-66.

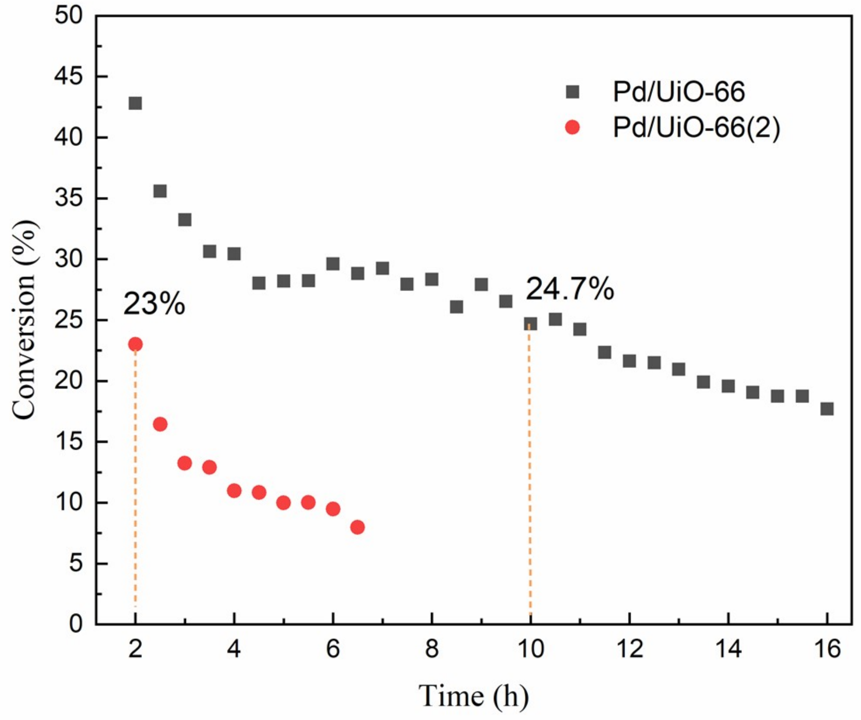

Fig. S5. The CO conversion of Pd/UiO-66, and Pd/UiO-66(2). Reaction conditions: CO/MN/Ar/N_2/NO =5/13.5/10/60/3, 120˚C, 0.1 MPa.

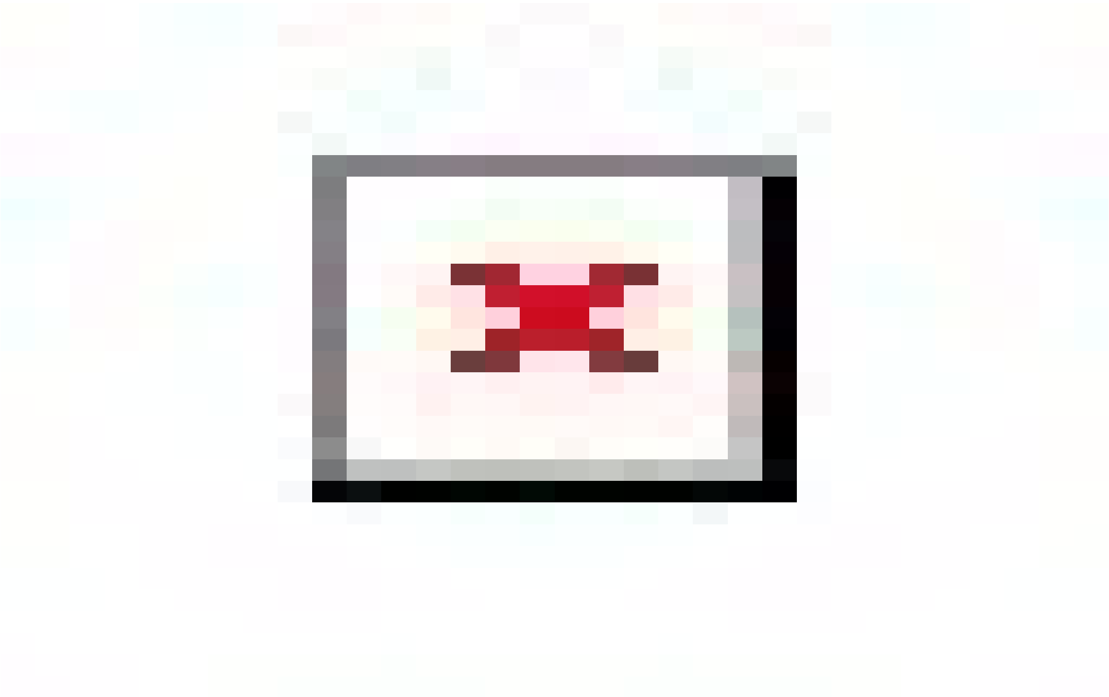

Fig. S6. The CO and MN conversion during the 70 h reaction of Pd/$Ce_{(0.1)}$-UiO-66.

Reaction conditions: CO/MN/Ar/N_2/NO =5/13.5/10/60/3, 120˚C, 0.1 MPa.

A catalyst stability test over 70 h has been performed for Pd/$Ce_{(0.1)}$-UiO-66, and the results are shown in Fig. S6. The CO conversion exhibited a similar deactivation trend within the first 11 h reaction, after which it decreased from 11 h to 25 h. It is interesting to find that the CO conversion began to increase after this decrease, reaching 25% at 33 h. After that, it decreased to 0 % at 67 h and remained nearly constant until 70 h. The increase in CO conversion is likely due to the damage to the BDC structure, which generated defects. Although the support remained the crystallization (Fig. S7), it was evidenced by the FTIR results in Fig. S8 that the band at 1689 cm^{-1} appeared and the band at 1656 cm^{-1} disappeared after the reaction. The appeared band was more obvious after 70 h of reaction, which was attributed to the emergence of the COOH vibration from terephthalic acid. The resulting structure increased the acidity of the support during the reaction, thus increasing the conversion of MN. The increased conversion of MN enhanced the concentration of the CH_3O* intermediate and briefly promoted the CO conversion [S1]. The role of surface acidity in promoting the conversion of MN is supported by the corresponding variation in MN conversion, which decreased from 46.1 % (2 h) to 21.3% (25 h) and subsequently

increased to 81.7 % (67 h). Moreover, according to the NH_3-TPD results in Fig. S9, the peak at about 250 ˚C that appeared after 70 h reaction might correspond to the formed structure.

The deactivation is primarily attributed to two concurrent processes involving migration and aggregation of Pd species. The first is the aggregation of Pd into larger particles reduced the concentration of accessible Pd species. This is supported by the STEM results of Pd/$Ce_{(0.1)}$-UiO-66 after 70 h reaction that large Pd particles were observed separated from UiO-66 particles, accompanied by a reduced Pd particle size within the support (Fig. S11 and Fig. S12). The second is the migration of Pd particles into the interior of the frameworks. As a result, the access of reactants to active sites was restricted by internal diffusion limitations, and meanwhile, Pd species might relocated to inactive positions during the migration process, which together contribute to the deactivation of the catalyst.

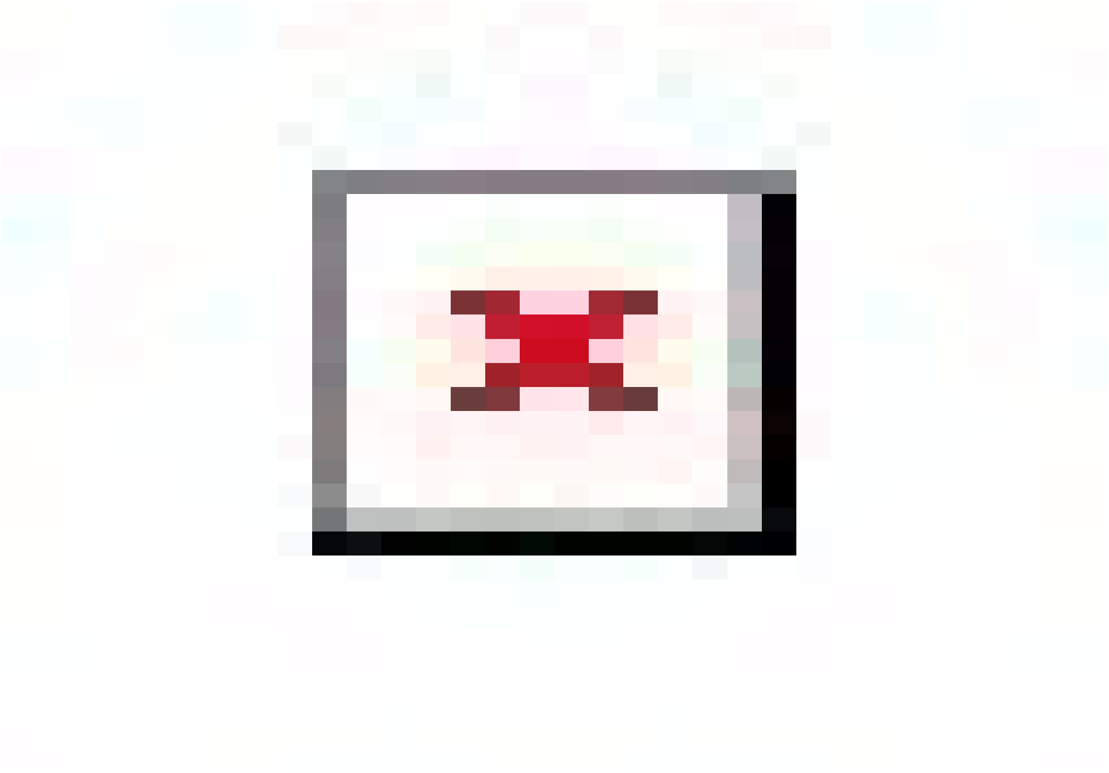

Fig. S7. XRD patterns of Pd/$Ce_{(0.1)}$-UiO-66 catalysts fresh, after 16 h and after 70 h reaction.

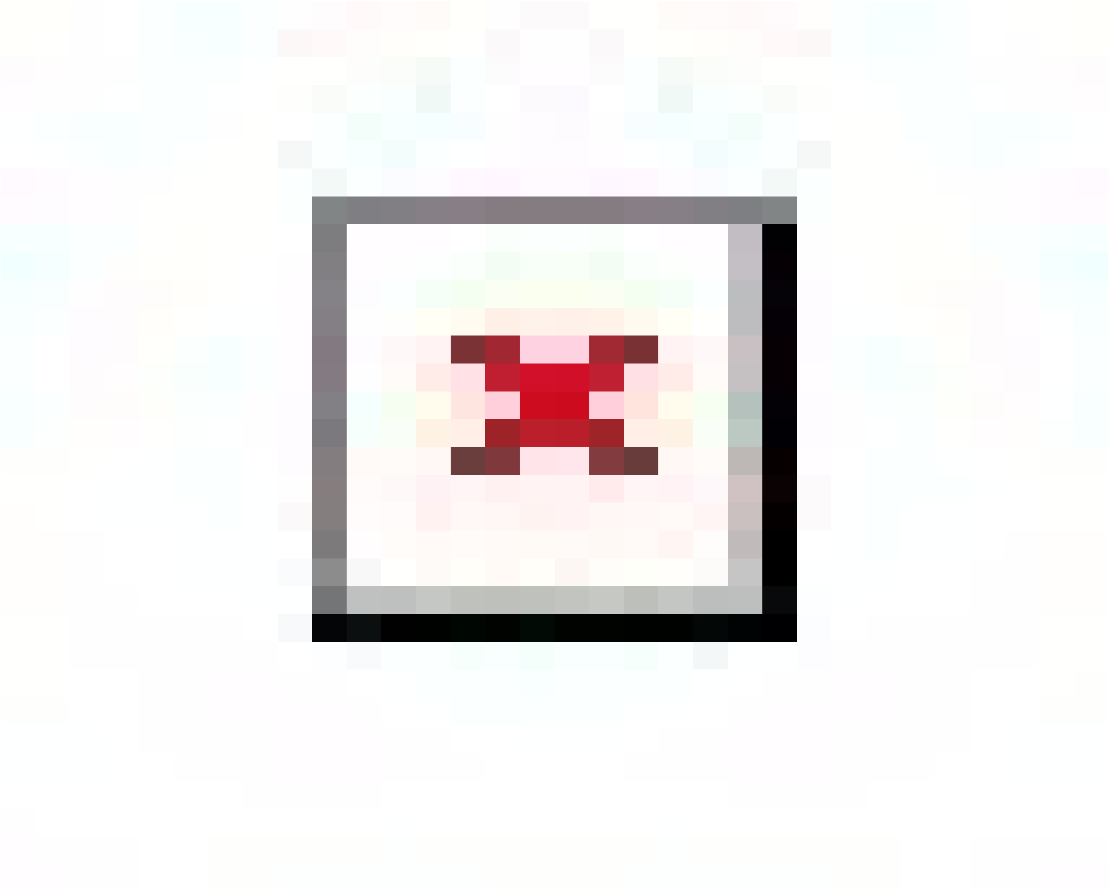

Fig. S8. FTIR spectra of Pd/$Ce_{(0.1)}$-UiO-66 catalysts fresh and after 70 h reaction.

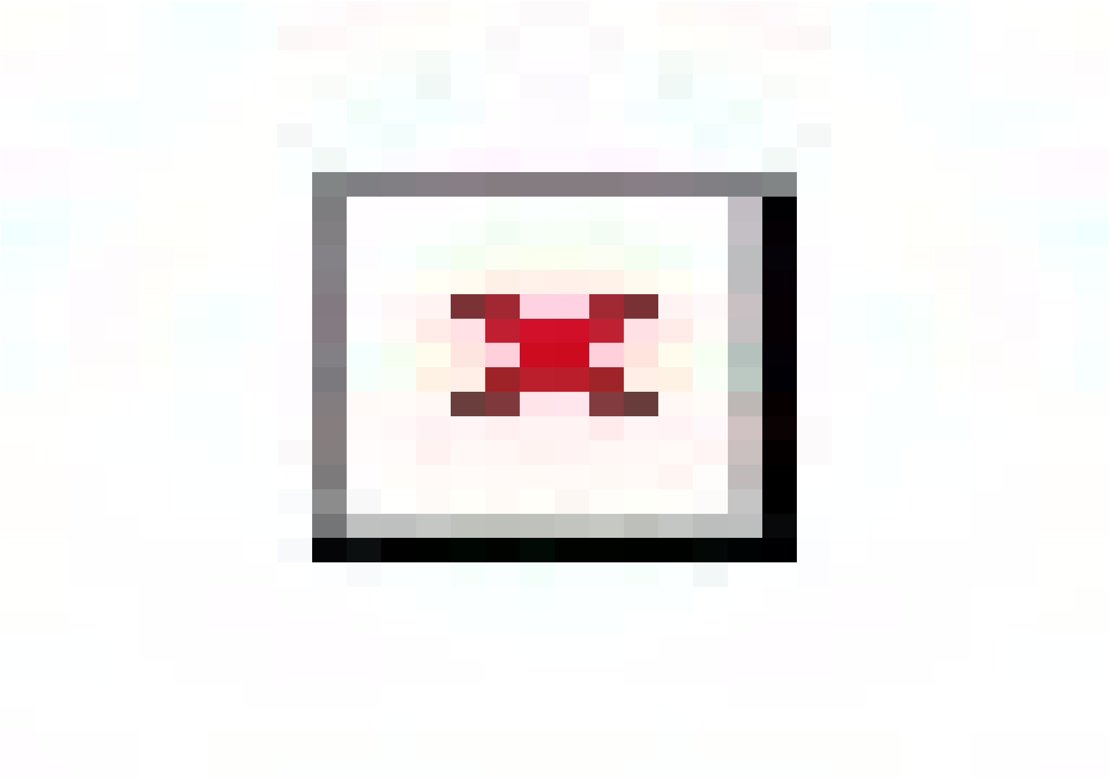

Fig. S9. NH_3-TPD profiles of Pd/Ce$_{(0.1)}$-UiO-66 catalysts fresh and after 70 h reaction.

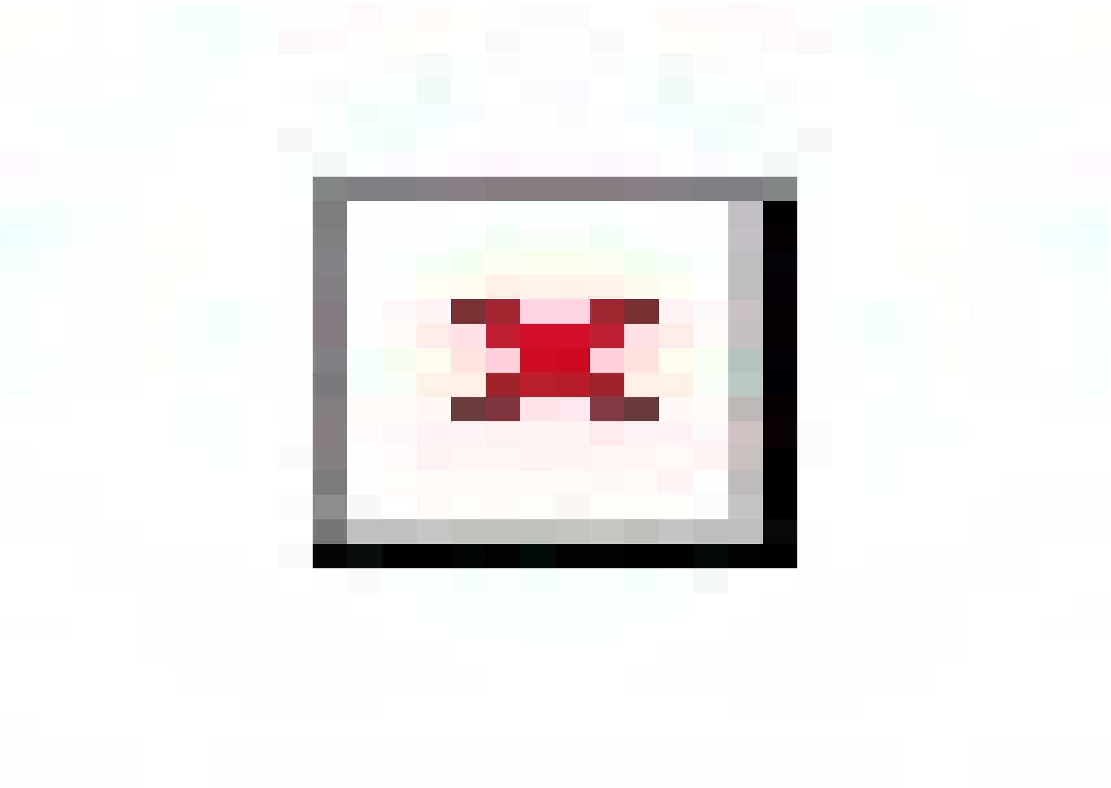

Fig. S10. (a, b) STEM image of Pd/$Ce_{(0.1)}$-UiO-66 fresh catalyst, (c-f) EDS elemental mapping of C, Zr, O, Pd.

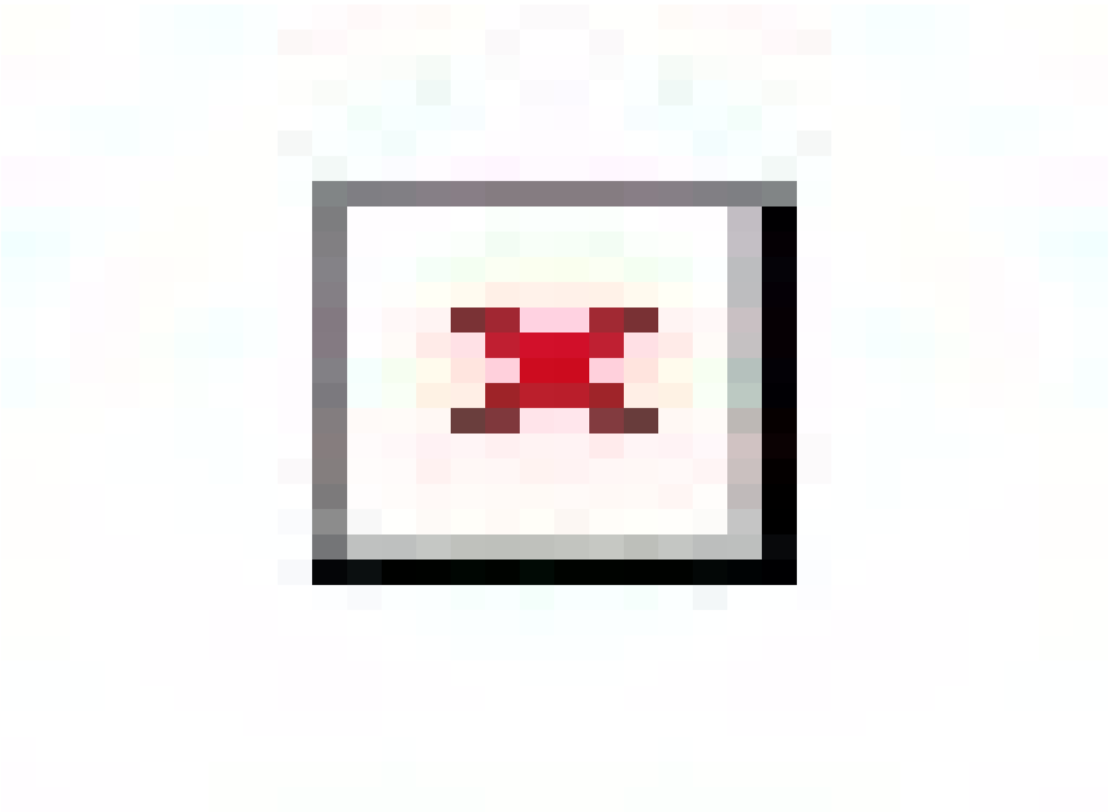

Fig. S11. (a, b) STEM image of Pd/$Ce_{(0.1)}$-UiO-66 after 70 h reaction, (c-f) EDS elemental mapping of C, Zr, O, Pd.

The distribution of Pd species of the Pd/$Ce_{(0.1)}$-UiO-66 catalyst before and after 70 h reaction were characterized by STEM and EDS. As shown in Fig. S10, Fig. S11 and Fig. S12, the results indicate that the concentration of Pd species decreased while remaining uniformly distributed as the reaction progressed. Meanwhile, it was observed that the particle size of Pd loaded on UiO-66 was slightly decreased, whereas some large Pd particles formed and separated from the carrier. These evidences collectively point to the migration and agglomeration of Pd.

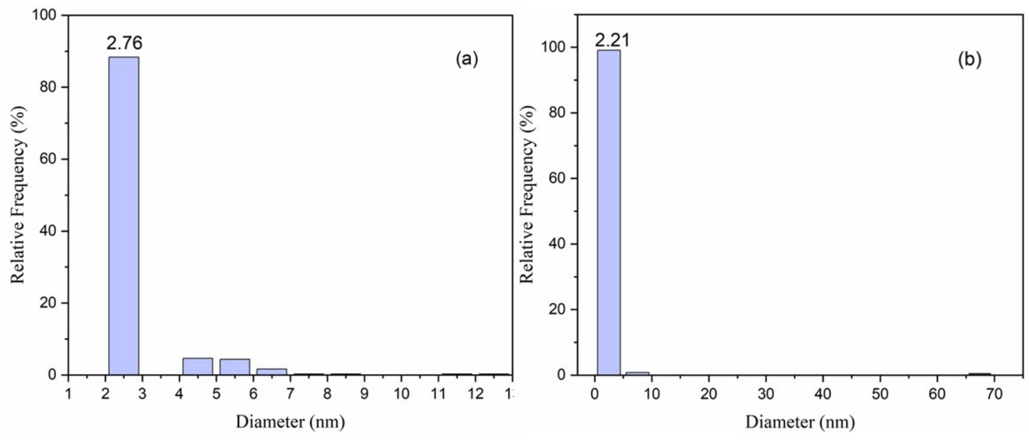

Fig. S12. The size distributions of Pd particles of the Pd/$Ce_{(0.1)}$-UiO-66 catalyst before (a) and after 70 h reaction (b).

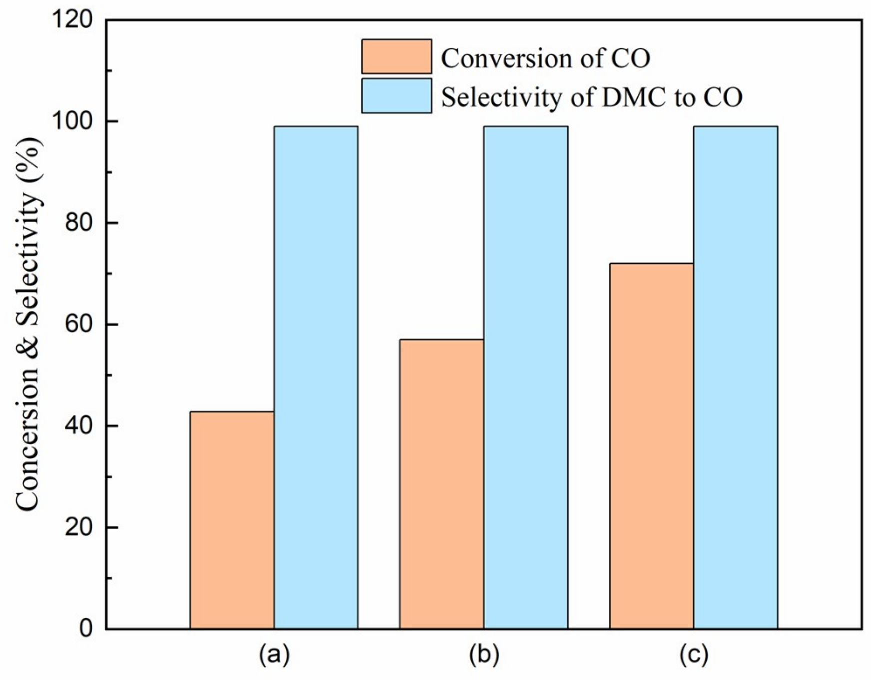

Fig. S13. The conversion of CO and the selectivity of DMC to CO in 2 h of (a) Pd/UiO-66, (b) Pd/UiO-66 + $CeCl_3$ (0.1), and (c) Pd/UiO-66 + $CeCl_3$ (0.2). Reaction conditions: CO/MN/Ar/N_2/NO =5/13.5/10/60/3, 120°C, 0.1 MPa.

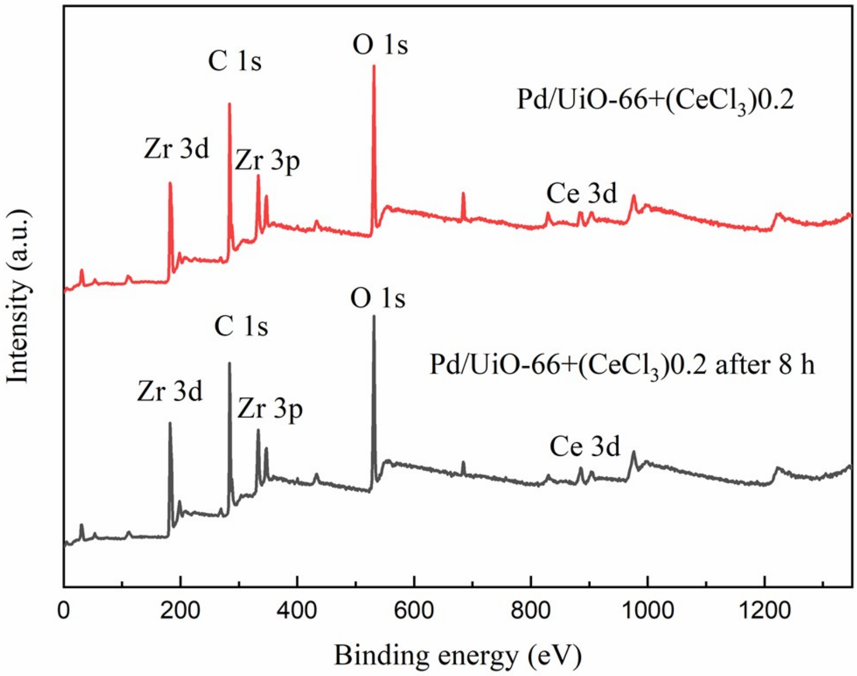

Fig. S14. XPS spectra of Pd/UiO-66 + $CeCl_3$ (0.2), and Pd/UiO-66 + $CeCl_3$ (0.2) after 8 h reaction.

Table S1 Surface area of UiO-66, $Ce_{(0.1)}$-UiO-66, $Ce_{(0.2)}$-UiO-66, and $Ce_{(0.5)}$-UiO-66.

Name	BET Area (m^2/g)
UiO-66	505.35
$Ce_{(0.1)}$-UiO-66	859.69
$Ce_{(0.2)}$-UiO-66	907.75
$Ce_{(0.5)}$-UiO-66	917.55

Table S2 Surface area of Pd/$Ce_{(0.1)}$-UiO-66 and Pd/$Ce_{(0.1)}$-UiO-66 after 8 h reaction.

Name	BET Area (m^2/g)	Pore Volume[a] (cm^3/h)	Average Pore Width (nm)
Pd/$Ce_{(0.1)}$-UiO-66	963.39	0.19	3.0
Pd/$Ce_{(0.1)}$-UiO-66 after 8 h	971.58	0.19	3.1

[a] the micropore volume determined by the t-plot method.

Reference

[S1] C.Z. Wang, B. Liu, P.Y. Liu, K. Huang, N.K. Xu, H.L. Guo, P. Bai, L.X. Ling, X.M. Liu, S. Mintova, Elucidation of the reaction mechanism of indirect oxidative carbonylation of methanol to dimethyl carbonate on Pd/NaY catalyst: Direct identification of reaction intermediates *J. Catal.* **2022**, 412, 30-41.
